# A Prognostic Model to Predict Risk of Dementia or Death in Older Veterans with Traumatic Brain Injury (TBI)

**DOI:** 10.1002/alz.092577

**Published:** 2025-01-09

**Authors:** Deborah E Barnes, Yixia Li, W John Boscardin, Amber L Bahorik, Carrie B. Peltz, Kristine Yaffe

**Affiliations:** ^1^ University of California, San Francisco, San Francisco, CA USA; ^2^ NCIRE‐The Veterans Health Research Institute, San Francisco, CA USA; ^3^ San Francisco Veterans Affairs Health Care System, San Francisco, CA USA; ^4^ Departments of Psychiatry and Behavioral Sciences, Neurology, and Epidemiology, University of California San Francisco, San Francisco, CA USA

## Abstract

**Background:**

Prior studies have found that traumatic brain injury (TBI) is associated with increased risk of both death and dementia in older adults including veterans. Clinically, it would be useful to be able to accurately predict the likelihood of these two outcomes for a given patient.

**Method:**

Our study population included all individuals aged 55 years or older who received care from the Veterans Health Administration between 2002 and 2019, had a TBI diagnosis, had at least one post‐TBI healthcare visit, and did not have a dementia diagnosis or medication before or up to 1 month after the TBI (n = 113,779). We used multinomial logistic regression with Lasso to develop a prognostic model that simultaneously predicts risk of dementia or death. Potential predictors included demographics, medical and psychiatric comorbidities, and healthcare utilization variables. Model accuracy was assessed based on c‐statistics. We also calculated observed outcome rates over decile of predicted risk and performed stratified analyses to assess for differences in accuracy based on TBI severity and combat veteran status.

**Result:**

Cohort members had a mean ± SD age of 68 ± 10 years; 6% were female; and 20% were non‐White or Hispanic. The final model included 22 variables. Key predictors included older age, being male, number of inpatient visits, and comorbidities including Parkinson’s disease, diabetes, weight loss, epilepsy, substance use disorder, and psychosis. The c‐statistics were 0.756 (95% confidence interval: 0.751, 0.761) for dementia and 0.783 (0.779, 0.786) for death. Model accuracy was slightly better for mild vs moderate/severe TBI (0.777 vs. 0.743 for dementia, 0.805 vs. 0.755 for death) and for combat vs. non‐combat veterans (0.776 vs. 0.751 for dementia, 0.806 vs. 0.776 for death). The observed proportion who developed dementia ranged from 3% to 43% over decile of predicted risk, while the observed proportion of deaths ranged from 4% to 64%.

**Conclusion:**

A single prognostic model that includes demographics, comorbidities, and utilization variables can predict risk of dementia or death in older veterans who have experienced TBI with good accuracy. Additional studies are needed to determine how this information might best be utilized to support better care.

